# Religion and Hate Crime Victimisation: A Representative Study of Young People in Finland

**DOI:** 10.1007/s43576-022-00079-6

**Published:** 2023-01-13

**Authors:** Sophie Litvak, Janne Kivivuori, Markus Kaakinen

**Affiliations:** Institute of Criminology and Legal Policy, University of Helsinki, Snellmaninkatu 10, PO Box 16, 00014 Helsinki, Finland

**Keywords:** Hate crime, Violence, Religion, Routines, Lifestyle, Victimisation

## Abstract

Key societal macro-trends, such as immigration and the increasing salience of post-secular and identity-based religiosity, are converging to increase the relevance of religion in everyday life. Such developments call for a reassessment of the religion–victimisation link. We analyse the prevalence and severity patterns of hate victimisation in different groups defined by religion and explore the links between routine activities and lifestyle factors in hate crime victimisation. Our research site, Finland, is a country with a long history of religious homogeneity, recently interrupted by religious pluralisation. We draw on the 2020 sweep of the Finnish Self-Report Delinquency Study (FSRD), a nationally representative crime survey targeting 15–16-year-olds (*N* = 5482). We found that religiously unaffiliated and Muslim youths have an above-average risk of hate crime victimisation. There were some indications that the patterns of victimisation are different across religious denominations. For instance, Muslim youths were more likely to be attacked by adults. Hate crime victimisation risk was not mediated by routines and lifestyles. Independent of religion, there was, however, a strong and direct positive association between hate victimisation and a risky lifestyle, i.e. substance use and interaction with delinquent peers. Comparing the findings with assault victimisation, we observed that the results are largely specific to hate crime offending rather than assault victimisation in general. We discuss the findings from the perspective of criminological theory, future research needs, and policy implications.

## Introduction

The role of religion is central and likely increasing in the contemporary world. In 2010, 84% of the world’s population were religiously affiliated. Projected global population trends, such as immigration and birth rate, indicate continued growth, with a great majority of the world's population expected to be religiously affiliated by 2050 (Stonawski et al., 2015). Extensive worldwide immigration, furthermore, contributes to the increasing pluralisation of the religious composition of countries and regions as previously religiously homogeneous areas become heterogeneous (Hackett et al., 2017).

Another vector of religious pluralisation is the increasing secularisation of developed and affluent societies. Though religious affiliation is expected to increase, in the current climate, young adults tend to be less religious than their elders. However, while this pattern is widespread, it is not universal and depends on the religion in question (Hackett et al., 2018). Though many parts of the world are secularising, this does not mean that the world as a whole is undergoing secularisation. On the contrary, the most religious areas of the globe produce the fastest population growth (Hackett et al., 2018). Whereas Christianity is predicted to remain constant throughout the next four decades, Islam is expected to have the fastest growth of all religions and establish itself as the second largest religion in the world (Hackett et al., 2017). Together, these trends imply that the overall global religious composition is changing, likely increasing the number and share of Muslims and persons who are switching or leaving their faiths (Stonawski et al., 2015).

The rising societal salience of religion also has repercussions for criminology, especially as the pluralisation of previously homogeneous societies increases the risk of hate crime in religiously defined subpopulations. This merits further research attention. In this paper, we define *hate crime* as a violent act in which the victims are targeted because of their language, skin colour, religion, societal opinions, or another similar characteristic. Hate offending is a type of violence that often targets minorities (Perry, 2002) and can be seriously harmful for communities and individuals alike (Hardy & Chakraborti, 2019). Aggression against an individual as a representative of a group makes the whole group a target of aggression. This can lead to fear and avoidance behaviour as an adaptation strategy, possibly leading to negative cycles when public space lacks capable guardians (Hardy & Chakraborti, 2019).

For individuals, hate crime victimisation can be a highly traumatic event; such types of victimisation have been linked to depression and suicide attempts among young people (Hardy & Chakraborti, 2019; Klomek et al., 2008). Prior studies found that hate crime victimisation has a greater impact on the individual’s physical and emotional well-being compared with victimisation that was not characterised by hate (Iganski & Lagou, 2014; Paterson et al., 2019). Some scholars claimed that hate crime “hurts” more, as it targets the core identity of the victim (Iganski, 2001). Religious affiliation and religiosity^1^ can trigger hate crime victimisation and lead to especially negative consequences in minority religious groups (Chakraborti & Garland, 2012).

Prior studies suggest that hate victimisation risk can is higher among youths (Enzmann et al., 2018) than among adults (Van Kesteren, 2016). This calls for research on hate crime among young people.^2^ Given the high cost of hate offending to individuals and communities and the rising social salience and increasing plurality of religions in contemporary societies, in this article, we study whether the risks of (violent) hate victimisation vary among young people from different religious groups, whether the patterns of hate victimisation differ across groups, and whether the possible differentials can be explained by routine activities and lifestyles.

## Routine Activities and Lifestyle Theories

The link between religious group identities and hate crime risk can be complex. At the individual level, religion can be a risk factor for hate victimisation, especially if a religiously defined group is perceived in an undesirable light by others (Rowatt & Al-Kire, 2021). However, religious belief and affiliation can also serve as protective factors against hate crime (Wallace Jr & Forman, 1998). Membership of a religious group could possibly increase the collective protection of the individuals within it from victimisation (Van Kesteren, 2016). Involving a set of beliefs, behaviours, and types of bonding and belonging, involvement in religious practices and monitored leisure activities could impact risks by influencing the time spent outside and engagement with risky routines (Sinha et al., 2007). This could lead to reduced exposure to criminogenic settings and, thus, to the possibility of becoming a crime victim. Clearly, elements of routines and lifestyles are pertinent in understanding the religion–victimisation nexus (Wallace Jr & Forman, 1998).

According to the routine activity theory (RAT), crime occurs when a motivated offender and a suitable target meet at the same time and in the same space without the presence of a capable guardian (Cohen & Felson, 1979). The lack of any of these three components is sufficient to prevent a crime from occurring (Felson & Cohen, 1980, p. 392). For instance, time spent outside of the home and away from the family is related to victimisation risk (Cohen & Felson, 1979, p. 591). Both the empty houses and the people who leave their “safe spaces” are exposed to motivated offenders, impacting victimisation risk. Thus, the routine activities approach has emphasised the influence of people’s location and movement in place and time on their victimisation risk.

In contrast, the lifestyle/exposure theory, first presented by Hindelang et al. in 1978, states that differences in lifestyle influence exposure to risky situations and, thus, affect a person’s victimisation risk. Lifestyles are shaped by personal characteristics, such as age, sex, race, income, employment, and marital status, as well as by expectations based on these qualities. What is expected of an adult is not expected of a child and vice versa. Accordingly, it is the person, his or her situation and social expectations that affect the experienced lifestyle. As a result of differential lifestyles, people differ in their propensity to become victims (Hindelang et al., 1978, p. 129).

In research practice, routine activities and lifestyle notions are often combined into a relatively amorphous general perspective (Pratt & Turanovic, 2016). Yet there are important differences. Routine activity theory involves a more general notion that crime can occur everywhere in the context of legal and legitimate routines, regardless of the riskiness of the situation and the victim’s association with risky behaviour. In contrast, the lifestyle approach (LSA) predicts that the riskiness of the activity is relevant to its potential to trigger victimisation. Therefore, while RAT focusses on the crime as an event in itself, lifestyle theory focusses on its context or riskiness (Pratt & Turanovic, 2016, pp. 335–337). Lifestyle theory (Hindelang et al., 1978) also suggests that spending time outside the home, especially at night, increases victimisation risk (Miethe et al., 1990; Felson et al., 2013), for it makes the victim more susceptible to the offender while putting them together in the same (possibly criminogenic) setting. We can summarise the difference between the approaches by saying that for LSA, the substantial content of activity matters more than the sheer patterning of legitimate activities in time and space. Going outside at night to take a cab to a friend’s house to watch a film together is not as criminogenic as going outside at night to drink alcohol with peers.

## Prior Research

There is a shortage of research specifically addressing the link between religion and hate victimisation in nationally representative samples. Comparing Switzerland and Finland, Staubli and Kivivuori (2017) observed that among Finnish adolescents, Muslim youths were at risk of experiencing more hate crimes and assaults than any other religious group (Staubli & Kivivuori, 2017, p. 5). In the preceding year, hate crime victimisation was higher among Finnish Muslims (24%) than among Swiss Muslims (7%), calling for more research on the Finnish scene. With reservations, an immigrant background could be taken into account while talking about religious pluralism. The study conducted by Van Kesteren (2016) showed that in all of the 14 observed countries, slightly more than a quarter of all victims of hate crime were immigrants. While belonging to a minority group increased the victimisation risk (Van Kesteren, 2016, p. 151), being single and young with an outgoing lifestyle and low income had a stronger effect on victimisation than immigration status (Van Kesteren, 2016, p. 150).

Generally, studies have aimed at including both risk-increasing and risk-reducing aspects of religion, but findings have been mixed. It has been argued that the religiosity of adolescents and the social attachment that goes with it increase the commitment to prosocial norms and induce levels of personal self-control while in a group (Khoury-Kassabri et al., 2015). This could mean that the religiosity of the (in this case Muslim) youngsters can serve as a protective/buffering factor against offending, and possibly against the victimisation that could accompany it. Examining the role of structured and unstructured activities on victimisation, Henson et al. (2010) suggested that structured activities (sport, homework, church, extracurricular) serve as a protective factor against hate crime victimisation, as they are to some extent monitored by parents and other adults. Unstructured activities (with peers or at work, depending on the work), away from home and parents, were suggested to increase the risk of hate crime victimisation.^3^ Nonetheless, the results did not corroborate this hypothesis. Ellonen et al. (2021) examined hate crime victimisation through individual and environmental predictors. Using data collected in 2016, hate crime victimisation was tested in relation to individual characteristics and social disorganisation theory. It was observed that a delinquent lifestyle was associated with hate crime victimisation. While not focussing on the religion–hate nexus, the study suggested that risk was linked to a high risk-taking tendency and a delinquent lifestyle rather than to other routines.

In addition to risk, the patterns and seriousness of hate crime may differ in different religious target groups. Thus, a UK study suggested that in hate crime, there is no “one-size-fits-all” pattern. While other hate crime was often committed by persons previously known to the victim, Muslims were more often victimised by unknown males in nightlife settings (Hardy & Chakraborti, 2019). These attacks were also described as more violent or sexual in nature than attacks conducted by females (Hardy & Chakraborti, 2019). That study, thus, points out the need to study not only risk but also the patterns and severity of victimisation.

Additionally, prior studies suggest that self-control is relevant in understanding the links between religion and victimisation. It is believed to be related to religiosity and religious affiliation. Studies suggest that religiosity can be a protective factor against impulsive/delinquent behaviour (Baier & Wright, 2001; Khoury-Kassabri et al., 2015) through its regulation of self-control. Furthermore, religious individuals who socialise with delinquent peers are less inclined to commit serious assaults than their non-religious peers. Religious beliefs and attachment can help youths resist the peer pressure of engaging in criminal behaviour (Massarwi et al., 2019; Desmond et al., 2008).

## Finland as a Research Context

Finland, a Nordic country with a population of 5.5 million, allows an informative study context of how religion is linked to hate victimisation. For centuries, nearly every Finn was born to the Evangelic-Lutheran Church, with a relatively small minority of Russian Orthodox Church members. These two denominations still have public law status and taxation right in Finland, even though anyone can exit them freely. This long historical background means that in Finland, as in the other Nordic countries, remaining in the national Church has been strongly based on shared tradition and culture rather than on individualistic religiosity (Tervo-Niemelä, 2021).

As in other Nordic countries, religious diversity within Finnish society has increased with immigration (see Kaakinen et al., 2018). The latest data on religious community membership from 2021 demonstrate that Christianity is the most prominent religion in Finland, accounting for 68% of the population (Statistics Finland, 2022). This is followed by individuals who are identified as non-religious (30%). People affiliated with other religious groups that are to some extend rooted in Christianity (such as Jehovah’s Witnesses and Latter-day Saints) make up the third largest group (0.38%), and people affiliated with Islam account for up to 0.38% of the Finnish population. All other religious groups (such as Eastern religions, Judaism, and neo-paganism) together make up less than 1 per cent.^4^ In addition, the numbers of those leaving the national Churches have increased during the twenty-first century, especially among young adults (Niemelä, 2015).^5^ On the face of it, this secular trend would appear to resemble a counter current to the globally increasing salience of religion. However, for some native citizens of affluent nations, the decision to leave the historical Church may involve motives other than a decline in personal religiosity. The main reason for resignation is the perceived conflict between personal beliefs and values and those represented by the national Church (Niemelä 2017, 2010 and 2015). Furthermore, some of those who decide to leave the church may describe themselves as “spiritual but not religious” (Seto, 2021). A majority of the population in Finland believes in God or in some divine force, while many distance themselves from Church doctrines (Kääriöinen & Niemelä, 2016). Thus, the diminishing popular basis of the de facto state Church does not only or necessarily involve secularisation or “atheism” (Niemelä, 2015).

According to an analysis based on crimes reported to the police and including all age groups, religion was the second most frequent target of police-recorded hate crime in Finland (13%), after ethnic or national origin. The most common types of hate crime were assaults, verbal insults, unlawful threats towards an individual belonging to national or ethnic minority group and conducted by a member of the majority group, and property damage (Rauta, 2022). According to police records, in 2021, 1026 cases of suspected hate offences in Finland were reported to the police, which indicates a 20% increase from the previous year. Out of this number of offences, 706 were filed by the police as hate crimes. Reporting on hate crime motivated by religious background increased by 23% from the previous year, and the most frequently victimised group were Muslims (Rauta, 2022).

In the third sweep of the International Self-Report Delinquency Study (ISRD), 5.1 per cent of Finnish 13–16-year-olds reported having been victims of hate crime during the preceding 12 months. The incidence of such victimisations per 100 persons was 16.5. In the total sample, the corresponding figures were 4.5% of youths and 16.6 incidents per 100 persons. Thus, the Finnish victimisation rate was close to the average in the 27 countries studied (Enzmann et al., 2018, p. 42). In terms of risk, Finland is a typical Western European country rather than an outlier.

In Finnish legislation, hate crime is not criminalised as a specific crime type. Rather, hate motives are considered to be aggravating circumstances influencing the severity of penal sanction (Criminal Code of Finland 6:5; Riku, 2019). According to the Criminal Code, *hate motive* refers to the commission of an offence based on the victim’s race, skin colour, birth status, national or ethnic origin, religion or belief, sexual orientation or disability or other corresponding identity (Juutinen, 2021). Thus, a hate crime can be any act that is defined as a crime by Finnish law.

## Research Questions

We focus on three groups defined by religion, Christians, Muslims, and young people describing themselves as non-religious youth. Here, Christians represent the mainstream affiliation in the national Church (Niemelä, 2015). Muslims and non-religious youth, in turn, represent religious minority groups that tend to be subject to different prejudices (Rowatt & Al-Kire, 2021). We examine the violent hate crime victimisation differentials of religious groups as reported by the victims, while the perceived motive of the aggressor is considered an empirical rather than definitional question. For social mechanisms, we explore whether the possible risk differentials can be explained by differences in routine activities and lifestyle factors. The specific research questions are: How prevalent is hate crime victimisation within different groups defined by religion? (RQ1) Are there pattern differences of self-reported hate victimisation between religious groups? (RQ2) Are religious affiliation and religiosity linked to hate crime victimisation, and, if so, in which direction (protective or risk factor)? (RQ3) Are the associations between religious affiliation and religiosity and hate crime victimisation mediated by routine activities and a risky lifestyle? (RQ4).

In addressing these questions, we add to prior research on several counts. We draw on a large nationally representative survey, thus complementing earlier city-based findings. We focus on the role of mediating mechanisms suggested by key theories, and by exploring the seriousness of the events. Finally, we supplement the research field on the crime–religion nexus by conducting research in Finland, a research site manifesting a recent societal shift from religious homogeneity to pluralism. This shift was caused by large-scale migration from the 1990s (Kaakinen et al., 2018; Skardhamar et al., 2014). At the same time, detachment from historical Church formations, sometimes signifying the heightened identity relevance of religious dogma, has accelerated within the native-born population (Kääriäinen, 2011). Importantly, while we speak about the victimisation risks of religious or religiously affiliated individuals, we by no means imply that the individuals hold any responsibility for the actions of the perpetrator.

## Data and Methods

Hate crime often remains unrecorded in official registers because victims do not report incidents to the police (Pezzella et al., 2019). The reasons for non-reporting include fear of retaliation, re-traumatisation, shame, and mistrust of the police (Van Kesteren, 2016: 141; Andersson et al., 2018: 78). We therefore opted to use a community survey to explore the full spectrum of the religion–hate nexus. The Finnish Self-Report Delinquency Study (FSRD-2020) is an anonymous crime survey targeting Finnish ninth-grade students (mainly 15- to 16-year-olds), with a nationally representative cluster sample of schools.^6^ The random sampling process was conducted by Statistics Finland. The data were collected in spring 2020 using online response during regular school hours under teacher supervision.^7^ The dataset includes 5,673 respondents with a 78% response rate (Kaakinen et al., 2021 and 2022). Half (50%) of the respondents were female, 48% were male, and 2% reported another gender. The vast majority (99%) of the respondents were between 15 and 16 years of age and the remaining minority between 17 and 18. The majority of the respondents were born in Finland (96%) and their mother tongue was Finnish (89%).

### Dependent Variable

We defined (violent) hate crime operationally with reference to the question included in the FSRD study. The question was: “Has anyone ever intentionally threatened you or targeted you with violence because of your language, skin colour, religion, societal opinions, or some other similar characteristic?” This question conforms to the definition of hate crimes as criminal acts against a person or group because of their specific identity features (Van Kesteren, 2016). The hate crime victimisation question uses the same wording as the third wave of the ISRD project. The use of the standard violent hate crime question additionally enables comparison with non-hate violence. Due to the relative rarity of the offence, we use a lifetime recall period in multivariate analysis.^8^

The FSRD additionally features follow-up questions for those who experienced the index crime during the previous 12 months. The main rationale for follow-up questions in self-report surveys is that they capture the nature of the events. Thus, we asked how many times the respondents had been victimised during the previous year. After that, they were asked to describe specific aspects of *the most recent offence*, tapping into seriousness. Thus, we enquired about the place of the crime, the number of offenders, and possible injuries sustained by the respondent. Regarding the offender, we asked the victim to describe his/her gender, age, national origin, and motives as they perceive them. One of the listed motives was religion. In describing the patterns of hate crime, we draw on these follow-up questions.

### Independent Variables

#### Religious Affiliation

Questions on religious affiliation and importance are derived from the standard ISRD questionnaire, developed by the ISRD group from sweep 3 (see Marshall et al., 2022). The respondents were asked, “What is your religion, or to which religion do you belong?” followed by a list of response options and the option to fill in the religion themselves if it was not listed. The religious groups that had fewer than 40 people in total (such as Pagan, Jewish, Mormon, and Eastern religions) were excluded from the analysis. The most common religiously defined groups were Christians^9^ (83%), followed by persons not affiliated or belonging to the major religious denominations (13%), and Muslims (2.4%). In the concluding discussion, we refer to some tentative differences between Sunni and Shia/other Muslims.

#### Importance of Religion (Religiosity)

The respondents were asked, “How important is religion for you personally?” The response alternatives ranged from 1 = not important at all to 5 = very important. In the multivariate analysis, we trichotomised this variable so that 1 = religion is not important at all, 2 = intermediate response categories, and 3 = religion is very important.

We use the labels “Christian”, “Christianity”, and “Non-religious” as shorthand^10^ expressions of self-defined group membership. Most of the respondents claiming no religious affiliation reported that religion had no importance in their lives; yet a significant minority (16%) within this non-affiliated group reported that religion (or perhaps spirituality, as discussed previously) had at least some importance. Therefore, the combination of religious affiliation and importance can draw a bigger picture of the role of the religion in young people’s lives.

#### Routines

Routines were captured by statements on leisure-time use. The items were *I spend time in public places with my friends after 9 pm, I return home on weekdays only after 10 pm*, *I go to parties where young people drink alcohol in the absence of adults*, and *I spend leisure time with older friends (not counting siblings).* Note that none of these items involve illegal behaviours per se*.* The Likert formatted response scale ranged from 1 = never to 5 = very often. For the multivariate analysis, we totalled and Z/2-scored the items (a z-scoring method where the variable is divided by two standard deviations instead of one; see Gelman, 2008). Cronbach’s alpha coefficient for the sum variable was 0.80 and the KMO (Kaiser–Meyer–Olkin) value was 0.77.

#### Lifestyle

To capture a risky lifestyle, we used a composite measure combining risky behaviours and differential association with delinquent peers. Risky behaviours included drinking alcohol to the point of drunkenness, driving under the influence of alcohol, cannabis use, and using drugs other than cannabis. Regarding delinquent friends, the respondents were asked if any of their friends had committed specific offences. The response options for questions about friends were 1 = none, 2 = one such friend, and 3 = more such friends. We used a composite measure of having friends who had used cannabis, shoplifted, participated in public fights, committed hate crimes offline, and/or harassed people sexually. For the multivariate analysis, we totalled and Z/2-scored the items (Cronbach’s alpha = 0.80 and KMO = 0.83).

### Control Variables

#### Gender

We used gender as a control variable. In our survey, 2% of respondents identified themselves as “other” gender. However, the “other” group was not included in the analysis due to the small group size.^11^

#### Birthplace

In our study, we asked the respondents to state the country in which they were born, presenting a list of options. If the birthplace did not appear on the list, respondents could add it manually. Fifty-seven countries were mentioned in the analysis. For the multivariate analysis, we dichotomised the birthplace into 0 = Finland and 1 = Elsewhere, in order to have the birthplace as a control variable for migration (status).

#### Self-Control

We additionally adjusted for self-control, as suggested by Pratt and Turanovic (2016, p. 347), as participation in risky activities could be a proxy for that individual trait. Self-control is a vital component in the lifestyle of youngsters, especially if they lead a risky lifestyle. However, since religiosity plays an important role in self-control regulation, we used self-control as a control variable. In the study, we used five statements to capture self-control. The items enquired about impulsivity, the inability to control delay of gratification, and risk-taking tendency. The Likert formatted response scale ranged from 1 = totally disagree to 4 = strongly agree. For the multivariate analysis, we Z/2-scored and totalled the items (Cronbach’s alpha = 0.82 and KMO = 0.80).

Missing data were not replaced. For the multivariate analysis, the missing values were omitted, leaving it with 5,429 valid cases. For the variable descriptors, see the Appendix.

### Analysis

We used descriptive comparison to examine whether the prevalence and patterns of hate crime varied by the victim’s religious affiliation. All of the differences between religious groups in terms of hate crime victimisation and event characteristics were analysed using cross tabulation and corresponding chi-square tests. We then moved on to the multivariate mediation analysis. Due to a dichotomous outcome, we used binary logistic regression. The mediation analysis has two purposes: to analyse whether the associations between hate crime victimisation and religious affiliation and religiosity are robust in the presence of controls and to examine whether possible differentials in hate crime victimisation risk are mediated by routines and lifestyle. We used the so-called KHB method and the corresponding Stata package developed by Karlson et al. (2011) to examine to what degree the total effect of religious affiliation and religiosity on hate crime risk is due to mediating factors (routine activities and a risky lifestyle). KHB is a decomposition method that accounts for the rescaling effect when comparing coefficients in nested nonlinear models. Furthermore, KHB allows for an analysis of direct and indirect associations in nonlinear models, similarly to the Sobel test often used for linear models (Kohler et al., 2011; Sobel, 1982).

Our analyses were conducted in two steps. Model 1 included religious affiliation groups (Christians as a reference category and Muslims and non-religious youth for comparison), religiosity (low religiosity as the base), and control variables (male, self-control, and foreign birthplace). In Model 2, routines and risky lifestyle were added as mediators. To explore the hate crime specificity of the findings, we conducted the same analyses using assault victimisation as the outcome. The purpose of these analyses was to ascertain whether the findings were particular to hate crime, rather than reflective of a general risk of victimisation. For our KHB models, we used a clustered (within schools) sandwich estimator for standard errors to account for our data structure. The estimation method used allows for an intragroup correlation for the standard errors, thus relaxing the normal regression assumption that the observations should be independent (see *estat vce* in StataCorp, 2021). Significance tests and confidence intervals were adjusted for design effects resulting from the cluster sample. We report goodness of fit by using pseudo-r^2^ coefficient for all models (Nagelkerke). We tested for potential multicollinearity and did not find excessive correlations between predictors (highest VIF was 1.67). We used STATA version 17 for the analyses.

## Descriptive Findings

### Prevalence of Hate Crime

Out of all respondents, 11% reported that they had experienced hate crime throughout their lives. Half of these (5%) reported that they had experienced hate crime in the past year (Table 1). There were notable differences in the risk of hate crime victimisation across groups defined by religion. Muslims had the highest overall lifetime rate of hate crime victimisation (41%).

**Table 1:** Hate crime victimisation by religious affiliation, %

	Any hate crime	
	Lifetime	Past year
Christians	8.5 (395)	4 (188)
Muslims	41.2 (56)	19.3 (26)
Non-religious	17.4 (123)	10.2 (72)
All	10.5 (574)	5 (286)
N	5482	5482
Chi2	193(2) < 0.001	102 (2) < 0.001

We additionally observed that the prevalence of lifetime hate crime victimisation differed among Sunni (47%) and Shia/other Muslims (35%). However, both sub-groups clearly experienced a higher prevalence of victimisation than Christians and non-religious young people

The non-religious youth also had an elevated hate crime risk. Of them, one-sixth had been victims of this type of crime (17%) throughout their lives. Their risk was, thus, on average twice as high as in the majority group of Christians (9%). When we examined whether there was any hate crime victimisation during the past year, similar differences between religious categories emerged.

### Patterns of Hate Crime

One of our research questions sought to explore whether the nature of hate crime events differs between religious groups. In this regard, the main findings are shown in Table 2. The Table compares the frequency, sustained injuries, victim-perceived motive^12^, and various offender characteristics by hate crime victims’ religious affiliation.

**Table 2:** Patterns of hate crime by religious affiliation of victims, % (*n*) of past year hate crime

	Christian	Muslim (all)	Non-religious	All	Chi^2^	*p*^a^
Incident						
Freq.per year						
Once	29 (53)	16 (4)	24 (17)	27 (74)		0.720
2–4 times	38 (70)	42(10)	42 (30)	39 (110)	2.087	
More than 5 times	33 (62)	42 (10)	34 (24)	34 (96)		
Injuries	15 (28)	23 (6)	24 (17)	18 (51)	3.237	0.198
Offender						
Male	81 (152)	85 (22)	72 (52)	79 (226)	4.230	0.376
Over 18	19 (35)	58 (15)	19 (13)	22 (63)	20.76	< 0.001
More than one	47 (88)	38.5 (10)	43 (31)	45 (129)	0.805	0.668
Motive						
Religious	6 (24)	23 (13)	4(5)	7.3 (42)	23.68	< 0.001
*N*	395	56	123	574		

^a^ *P ≤ 0.05 **P ≤ 0.01 ***P ≤ 0.001 *ns*  not significant

Overall, few statistically significant differences emerged. We found that Muslims were more likely to have been aggressed against by an adult and that the incidents were more often religiously motivated, as perceived by the victim. Regarding offender characteristics, irrespective of the victim’s religious affiliation, on average 79% of the perpetrators were male. Most groups had experienced hate crime attacks from peers. However, 58% of all Muslim hate crime victims were aggressed against by people older than 18. Regarding victimisation motives, Muslim victims of hate crime attacks more often (23%) attributed the motive for the inflicted attacks to their religious affiliation. In other words, the hate crime attacks against Muslims were more often motivated by their religion than the hate crime attacks on other groups.

These differences tentatively suggest a more serious type of hate crime against Muslims. Generally, the patterns of victimisation appeared to be quite similar between Christians and non-religious youths. This indicates that the observed differences capture patterns specific to Muslims. We found no statistically significant differences in terms of incident frequency, injuries, gender, and multiple offenders.

We also inspected the role of various places in hate victimisation patterns, as there were no a priori grounds for seeing one of them as the most “serious”. We observed that in total almost half (45%) of all hate crime incidents took place at school. While only marginally significant, this analysis tentatively suggests that Muslim youths may be more likely to experience hate crime on the street (35% compared to 17% for other groups [chi^2^ = 9.03, *p* = 0.060]). Additionally, we observed that Muslim victims had the highest frequency of severe injuries that required medical assistance (11% compared to 4% for non-religious, and 2% for Christians; not reported in Table 2) as a result of a hate crime attack. Although these differences in severe injuries were statistically significant (chi^2^ = 9.60, *p* = 0.048), the assumption of the minimal cell size for expected observations was not met (minimum of five observations; see McHugh, 2013). Regarding the victim’s gender, it appeared that across all of the observed religions, males were more often victimised (57%) through hate crime than females (43%) and that those differences were significant (chi^2^ = 13.36, *p* < 0.001).

## Multivariate Analysis

According to our multivariate models (Table 3), Muslim affiliation (OR = 4.48, *p* < 0.001) and non-religious affiliation crime risk (OR = 1.72, *p* < 0.001) were linked to an elevated hate crime risk when compared to the Christian majority, net of other examined correlates (Model 2 in Table 3 below). The high importance of religion was also associated with hate crime risk (OR = 1.90, *p* < 0.001). Routine activities were not linked to hate crime risk (OR = 0.90, *p* = 0.40), whereas lifestyle factors were (OR = 3.33, *p* < 0.001).

**Table 3:** Logistic regression models of hate crime victimisation

		Hate crime		Assault	
		Model 1	Model 2	Model 1	Model 2
Religious affiliation	Non-religious	1.857 (4.56)***	1.719 (3.77)***	1.270 (2.25)**	1.164 (1.37)
	Muslims	4.573 (7.85)***	4.481 (6.47)***	1.082 (0.35)	0.961 (−0.16)
Religiosity	Moderate	0.875 (−1.34)	0.924 (−0.75)	0.911 (−1.33)	0.964 (−0.48)
	High	1.700 (3.42)***	1.900 (3.86)***	1.126 (1.07)	1.274 (1.96)*
	Male	1.232 (2.50)*	1.141 (1.40)	2.130 (9.58)***	2.085 (8.89)***
	Born abroad	3.161 (6.06)***	3.658 (6.43)***	0.960 (−0.21)	1.040 (0.19)
	Self-control	2.242 (8.93)***	1.333 (2.51)*	2.216 (13.31)***	1.413 (4.60)***
	Routines		0.903 (−0.83)		0.774 (−3.19)***
	Risky lifestyle		3.330 (9.82)***		3.824 (15.56)***
	Nagelkerke r2	0.07	0.12	0.05	0.10

^b^N = 5329. Reference category in affiliation = Christians. Reference category in religiosity (importance of religion) = low. Gender (male) treated as dichotomous (“other” excluded from analysis). Exponentiated coefficients; t statistics in parentheses *p < 0.05, **p < 0.01, ***p < 0.001. The same models are shown for assault victimisation for comparison^*b*^

Some interesting findings emerged when the mediating mechanisms were in focus. First, entering routine activities and lifestyle left the significant main effects of religious affiliation and importance intact. The identity status of the person and the internal relevance of religion did not lose significance when their routines and lifestyles were factored in. We did not find any significant indirect effects as the routines and risky lifestyle did not mediate the association between the risk of hate crime victimisation and Muslim affiliation (*p* = 0.30), non-religiousness (*p* = 0.40), moderate religiosity (*p* = 0.52), or high religiosity (*p* = 0.26). This suggests that the hate crime victimisation risk was, to a relevant degree, independent of such mechanisms. This, in turn, could reflect the fact that the offenders were motivated to target members of religious groups outside of standard youth delinquency contexts, also aggressing against victims solely because of their known or presumed identity. Other mechanisms could also be involved, such as the visibility of the religion of the victim (which was not measured here).

We also explored the interplay between routines and lifestyles to test whether they would cancel each other out. There was a significant positive association between routines and the risk of hate crime victimisation (OR = 1.73, *p* < 0.001) before adding risky lifestyle to the model. When both routines and risky lifestyle were simultaneously added, the association was no longer significant (Model 2). This implies a confounding or mediating effect between routines and lifestyles.

### Comparison with Assault

With regard to the comparative analysis using assault as an outcome, the results mostly indicated that the religion variable links are specific to hate crime. For instance, looking at general victimisation prevalence rates, non-religious youths had the highest figures, showing a different pattern. In pattern analyses, there were more similarities, with Muslims typically showing a more serious victimisation pattern in both types of incidents. Muslim victims seemed to have a more adult and street-oriented aggressor pattern in both hate crime and assault victimisation, while the other two groups were closer to a school and youth-based victimisation pattern. For the multivariate analysis, we also show the final model for assault victimisation (Table 3, Model 2). The main finding is that religious affiliation, and the importance of religion are not linked to assault risk. Thus, the association of religiously defined group membership with hate crime is hate-specific, not a general crime risk-related finding. Moreover, we found the indirect association between religious affiliation and religiosity and hate crime victimisation to be non-significant.^13^

## Discussion

### Main Results

As regards descriptive findings, we observed that Muslims had the highest prevalence of hate crime victimisation irrespective of recall period length. More than 40% of Muslim youths had been victimised by hate crime at least once in their lives.

We found that the group self-defining as non-religious had an elevated risk of hate crime victimisation. More than one in six in this group had been a victim of hate crime at least once in their lives. This group mostly includes young people who have exited the Finnish Evangelical Lutheran Church, or whose parents have done so.^14^ This group likely includes youths who are “spiritual but not religious” (SBNR) and may be as religious as some in the majority “Christian” group, yet without church membership as a part of their religious identity. The elevated hate victimisation risk in this group adds to recent research observing above-average offending rates among the “spiritual but not religious” youth (Seto, 2021).

We also explored the patterns of the hate incidents across groups defined by religious affiliation. We observed that Muslim youths were more likely to be attacked by adults, and the victim-perceived motive was more likely to be religion. Generally, we found tentative indications that Muslims may have a hate victimisation profile that is more oriented to street-based incidents committed by older persons and possibly resulting more often in injuries. In contrast, non-religious young people appeared to be more at risk at school, possibly in situations resembling school bullying; this street vs. school pattern of victimisation should be explored in the future research. The findings are consistent with Perry’s observations that Muslim minorities are subjected to verbal hate incidents in public by older generations (Perry, 2016, p. 218). Generally, we detected that hate crime against Muslims can be more serious than hate crime against Christian or non-religious groups. However, the non-religious minority sustained injuries as often as the Muslim youths. Hence, their experiences can also be considered serious.

Inspired by routine activities and lifestyle theories, we explored whether the impact of religion on hate crime victimisation risk is mediated by the victims’ routine activities and lifestyle. We did not observe such mediation. We conclude that hate crime victimisation risk is, to a relevant degree, independent of victim routine activities and lifestyles. It is possible that the offenders are motivated to target members of religious groups outside the traditional risk contexts of youth delinquency. Other mechanisms can also be involved, such as the visibility of the religion of the victim. This should be examined in the future research.

To explore the generality vs. speciality of our hate crime-related findings, we reran the analyses by changing the outcome to assault victimisation. The findings suggest that hate victimisation is not simply a reflection of a general victimisation and that the role of religion in explaining risk differentials is probably more relevant than can be judged on the basis of victims’ responses regarding offender motivations. As for descriptive findings, non-religious individuals had the highest risk of assault victimisation. Yet, seriousness patterns were more similar across offences. Muslim youth had a more street and adult offender-based pattern, with more frequent injuries, in both assault and hate crime victimisation. The multivariate analysis corroborated that religious affiliation and religiosity were linked to hate victimisation but not to assault victimisation, reflecting the specificity of hate crime.

### Theoretical Implications

The descriptive findings of this research suggest that, in the future, hate crime studies should focus on the seriousness and the physical/mental severity of incidents in addition to their patterns and prevalence. Groups defined by religion differ in hate crime victimisation prevalence, but they also differ in the severity of incidents against them. Our findings additionally suggested that routine activities and risky lifestyles do not mediate the influence of religion on hate crime victimisation. In other words, in this analysis the link between religion and hate victimisation appears to be direct. This type of crime victimisation seems to be relatively disconnected from victim participation in risky lifestyles and delinquent networks.

Pratt and Turanovic (2016) have suggested that what is actually being done may matter more for crime victimisation than simply being out of the home at specific times. This is in line with our additional finding that the effect of routines (unsupervised leisure) was no longer a significant predictor of hate crime victimisation after adding the lifestyle factors to our models. The specific activities increasing the risk of victimisation calls for further study.

Needless to say, there could be other mediators besides those we have explored here. To overcome the limitations of the current study, several constructs call for attention in the future research using representative or large survey datasets. One candidate is the visibility of the religion. The use of visible religious insignia, such as clothes or symbols, could partially explain the link between victimisation and lifestyle/routines. This is why it was added to the ISRD-4 instrument at our initiative, an analytic option we plan to pursue. Previous research showed that the salience of a religious affiliation might put the victims in a position that the offender might see as vulnerability (Hardy & Chakraborti, 2019). The same applies to language spoken in public places, as this can help motivate offenders in target selection. Thus, Perry (2016, p. 217) has observed a connection between religious Muslim holy days and anti-religious incidents. During religious holy days and rituals, religious Muslims tend to dress in a traditional way, which increases their visibility and positions them as suitable targets.^15^ The festive connection may be particularly triggering if offenders consider Muslims to be “taking over” and want to “set the record straight” (see also Andersson et al., 2018).

The purpose of this study was to examine the hate crime victimisation risk of religious groups, irrespective of perceived offender motivation. Indeed, relatively few victims perceived the most recent incident as being religiously motivated. The reliability of asking victims to assess offender motivation may be limited. Notably, it could reflect the intersectional nature of the social disadvantage suffered by specific groups, combining minority religious status with immigration or another similar background. The offenders may also have multiple motives (Andersson et al., 2018), calling for a focus on the offender perspective in hate crime studies.

Based on this, it is clear that our measure of routines also lacks some relevant dimensions, such as visits to religious places of worship like mosques, synagogues, and churches (McNeeley & Overstreet, 2018). Finally, future research should have more information about the immediate foreground of the index crime, such as whether the victim was alone or in company. Target availability heuristics can be differentially triggered in motivated offenders depending on estimates of retaliation by co-religionists and neutral third parties. These aspects will be addressed in the next stages of our project.

### Limitations

Regarding the limitations of this research, standard caveats related to the use of cross-sectional data apply, including the temporal order of the factors included in the mediation analysis. The Covid-19-related school closures during the data collection in spring 2020 are unlikely to have impacted the validity of analysis (Kaakinen et al., 2022). The use of self-reports makes our analysis independent of national law and therefore transferable to other jurisdictions. In addition, the use of self-report measures enables us to analyse those incidents that were not reported to authorities (the great majority of cases). However, it also means that the responses rely on the subjective views of the respondents. To a degree, differences in victimisation between religion-based groups could reflect differences in the sensitivity to perceive conflicts as “criminal” or “violent”. It is possible that the religiosity of the person cues him or her to project religious motives to general minority-targeted attacks. On the other hand, our findings suggest that religious groups may also downplay the role of religion in hate crime targeting, and reliance on motive-based definitions might underestimate true group risk differentials. Nevertheless, religious group differentials were substantial irrespective of the religious motive attribution. The increasing societal relevance of religion can be reflected in the individual frames of interpretation concerning action motives.

Future research should attend to religious pluralism in a more nuanced manner. Crime-religion surveys need measures capturing variation within general categories like “Christians” and “Non-religious”. Another facet of pluralism is the sheer number of (old and new) religious communities. Thus, we were unable to analyse several religious groups due to the small number of persons from those groups in our sample. Such small groups include at least members of the Jewish community, sub-categories of Islam, members of Eastern religions, and neo-pagan groups. For instance, the possible differences between Sunni and Shia/Other Muslims deserves attention, as the former may suffer from more serious hate victimisation in the Finnish context. Finally, future studies could expand from routines and lifestyles to the “causes of the causes”, such as social structural factors.

Taken together, future research on the religion–victimisation link should draw on large international samples. Other forms of hate crime than interpersonal violence should be addressed as well. To name just one example, hate crimes can be directed towards the religious/communal institutions or places of worship that are important to the member(s) of the targeted group (Ulmer & Scheitle, 2020). Furthermore, more qualitative research is needed in order to conduct in-depth analyses of mechanisms.^16^ In particular, our finding that youths identifying themselves as non-religious have an above-average hate victimisation risk calls for qualitative research. For affluent countries, and possibly for all kinds of countries, studies should be sensitive to the new and often identity-salient uses of religious experience to avoid equating rising non-affiliation with the irrelevance of religion. In today’s post-secular, post-materialist, and individualist society, measuring the religion–crime link requires increasingly nuanced attention to measurement.

### Policy Conclusions

The findings on prevalence and patterns suggest that hate crime prevention is most acutely needed among the Muslim population. It is, however, of policy interest that the non-religious group also suffered from elevated hate crime risk. Any religious deviation from the majority identification appears to carry some risk of hate crime victimisation. Due to the larger number of non-religious youths in the population, there are actually more non-religious hate crime victims than Muslim victims. However, Muslims may suffer from qualitatively more serious types of victimisation, such as being victimised by older persons.

Religious tolerance should be taught to majority populations, as well as to minority religious affiliations. For example, campaigns on religious tolerance at school, or posters about civil courage (“if you see something, say something”) to tackle hate crime on the streets, could be tried and evaluated in research. For the purposes of crime prevention, it is crucial to understand where hate crime incidents occur, against whom, and by whom. Here, we observed, for instance, that crimes against Muslims tend to be street-oriented, while crimes against non-religious youths are more often school-related. Knowing the exact patterns will be helpful in developing strategies for religious hate crime prevention.

## Data Availability

All data used in the study will be made fully available via the Finnish Social Science Data Archive in 2023. Before that, data will be available on reasonable request.

## Appendix

Variable descriptors used in this study.

**Table:**

	*Min*	*Max*	*Mean*	*SD*	*N*
Dependent Variable					
Hate crime victimisation in a lifetime	0	1	0.109	0.312	5673
Demographics					
Age	15	18	15.24	15.24	5657
Male	0	1	0.494	0.500	5561
Born abroad	0	1	0.041	0.198	5654
Self-control					
I often act on a whim	1	4	2.468	0.818	5533
I do anything that makes me happy, even if it hinders the achievement of long-term happiness	1	4	2.086	0.853	5533
I like to test myself every now and then by doing something a little dangerous	1	4	2.079	0.934	5532
Sometimes I take risks just for fun	1	4	2.173	0.962	5532
Excitement and adventure are more important to me than safety	1	4	2.054	0.879	5532
Religion and personal importance thereof					
Non-religious	0	1	0.128	0.334	5482
Christians	0	1	0.846	0.360	5482
Muslims	0	1	0.024	0.154	5482
Personal importance of religion	1	5	1.843	1.099	5578
Routines					
Being with friends in public places after 9 pm	1	5	2.631	1.202	5606
Not coming home on weekday evenings until after 10 pm	1	5	1.991	1.028	5606
Going to parties at which many young people drink alcohol and adults are absent	1	5	1.857	1.126	5606
Spending free time with older friends (not counting siblings)	1	5	2.270	1.228	5606
Lifestyle					
Ever drank alcohol and felt drunk?	0	1	0.413	0.492	5673
Ever driven drunk?	0	1	0.084	0.277	5673
Ever tried/used marijuana or hashish?	0	1	0.091	0.287	5673
Ever tried/used drugs OTHER than marijuana or hashish?	0	1	0.039	0.195	5673
Friends					
Friends that used marijuana or hashish	1	3	1.557	0.801	5589
Friends that stole from a shop or kiosk	1	3	1.759	0.827	5589
Friends that fought in a public place	1	3	1.508	0.752	5589
Friends that harmed another people based on their belonging to a certain group of people	1	3	1.112	0.398	5589
Friends that sexually harassed me or sexually harassed another person	1	3	1.154	0.457	5589

SD = Standard Deviation.

## Footnotes

1 By religiosity we refer to the personal importance of religion.

2 Van Kesteren argued that young people are more exposed to hate crime because of their possible environment in the night-time economy in which they might be exposed to their perpetrators (Van Kesteren, 2016, p. 155).

3 According to previous studies, unstructured routines and socialising also increase delinquency levels (Osgood & Anderson, 2004) and were linked to violent behaviour (Maimon & Browning, 2010).

4 Statistics on religious community membership probably underestimate the number of persons identifying as Muslim.

5 This Church, whose members were once nearly entirely Finnish, and the smaller Orthodox Church in Finland, have public law status with a taxation right. Persons exiting these Churches may, therefore, also have economic motives for doing so.

6 Originally developed as an offshoot of the International Self-Report Delinquency (ISDRD) project, the FSRD has many similar questions, allowing for comparisons. Note, however, that the ISRD is a city-based sample, while the FSRD is nationally representative.

7 During the Covid-19-related school lockdown, 14% of the sample responded during remote teaching at home, but the classes were still supervised online by the teachers.

8 This recall period is also less susceptible to possible Covid lockdown impacts that may affect routines and lifestyles.

9 Includes Christian movements such as Laestadians and Pentecostalists, some of whom may not be members of Finland’s Evangelical Lutheran Church.

10 Christian, Christianity, Catholic, and Protestant, and similar, were categorised under Christian affiliation, Agnostic, and Atheist under non-religious.

11 The data show that many people with non-binary gender identity suffer from hate crime. Although this is an important issue to study, it is not the focus of this paper.

12 The FSRD questionnaire includes a question on how the victim perceives the main motive for their victimisation.

13 The p-value was 0.33 for Muslims; 0.36 for non-religious; 0.55 for moderate religiosity; and 0.27 for high religiosity.

14 In Finland, a person who has been a member of the majority Evangelical Lutheran Church since birth (as a result of parental affiliation) can exit the church at the age of 15 with parental consent and from the age of 18 without consent.

15 *Suitable target* is a technical term in the routine activity theory and does not have moral or evaluative implications.

16 The research that was conducted by Hardy and Chakraborti in 2020 provided very useful new information on the mechanisms and patterns behind hate crime. However, they focused on hate crime as a whole, without a specific focus on crimes motivated by hatred towards the religion of the victims.
